# Brain Work and Skull Growth

**Published:** 1879-08

**Authors:** 


### BRAIN WORK AND SKULL GROWTH.

The London *Medical Record* sums up as follows the results of some very interesting measurements of heads by two French physicians, Messrs. Lacassagne and Cliquet:

Having the patients, doctors, attendants, and officers of the Val de Grace at their disposal, they meaured the heads of one hundred and ninety doctors of medicine, one hundred and thirty-three soldiers who had received an elementary in, struction, ninety soldiers who could neither read nor write- and ninety-one soldiers who were prisoners. The instrument used was the same which hatters employ in measuring the heads of their customers; it is called the conformator, and gives a very correct idea of the proportions and dimensions of the heads in question. The results were in favor of the doctors; the frontal diameter was also much more considerable than that of the soldiers, etc. Nor are both halves of the head symmetrically developed: in students, the left frontal region is more developed than the right; in illiterate individuals, the right occipital region is larger than the left. The authors have derived the following conclusions from their experiments: 1. The heads of students who have worked much with their brains are much more developed than those of illiterate individuals, or such as have allowed their brains to remain inactive. 2. In students the frontal region is more developed than the occipital region, or, if there should be any difference in favor of the latter, it is very small; while in illiterate people the latter regions are the largest.

